# Hospital Accounts

**Published:** 1893-04-15

**Authors:** 


### The Institutional Workshop.

### HOSPITAL ADMINISTRATION.

### HOSPITAL ACCOUNTS.

It is matter for congratulation that each annual meeting of Governors of the larger hospitals throughout the country proves that the authorities are now alive to the importance of adopting a uniform system of accounts. In *Burdett's Hospital Annual* an attempt has been made to compare the relative cost per bed, and also the actual cost of administration at every class of hospital, metropolitan and provincial. The difficulties which the Editor had to contend with, owing to the different systems of accounts which prevail at the present time at provincial, Scotch, and Irish hospitals, were so great as to prove almost insurmountable. With the co-operation of the Secretaries, however, most of the difficulties have been overcome, and the correspondence which has arisen in consequence has led the committees to reconsider the form of accounts in several of the larger provincial institutions.

Thus, at the annual meeting of the Birmingham General Hospital, attention was drawn to an error in *Burdett's Hospital Annual*, which arose from the manner in which the accounts were published in the annual report. It appeared that the salaries rightly chargeable to the management seemed higher than they really were, and so the management expenses at the General Hospital, Birmingham, were said to be something like 17 per cent., whereas, when all the items of expenditure were properly classified they proved to be under 8 per cent. In calling attention to this error, the speakers at the annual meeeting intimated that the Finance Committee, it was understood, would be asked to reconsider the form in which the accounts were kept, so as to bring them more into accord with those of other large hospitals, in order that one could easily be compared with the other. A letter was received from the editor of the *Annual* expressing his regret at the error which had occurred, and we hope all will realise that however regrettable such errors may be, they sometimes lead, as in the present case, to important reforms. No doubt the accounts of the Birmingham General Hospital will henceforth be kept upon the uniform system adopted by the Council of the Metropolitan Hospital Sunday Fund after conference with the Committee of Hospital Secretaries.

The Report of the Royal Infirmary, Newcastle, 1892, contains the accounts of the institution printed in the same form as they have appeared for upwards of 100 years. In addition to this the House Governor, Mr. Redmayne, has published, with the consent of the committee, an income and expenditure account on the uniform system above referred to. These new accounts only occupy two pages of the Report, but they place the Royal Infirmary, Newcastle, in the important position of being able to compare its expenditure with that of the most reputable institutions, and so to ascertain how far, if at all, the sources of income and the items of expenditure differ at one institution as compared with another.

We look forward to the time when every hospital of repute will adopt this same system of accounts. Meanwhile it is satisfactory to have the evidence of one of the ablest members of the staff of a leading financial paper to the effect that having closely investigated the accounts of all the special philanthropic institutions in Great Britain, he has arrived at the conclusion, that on the whole, hospital accounts, now so generally kept upon the uniform system, leave little to be desired. On the other hand, the accounts of missions, orphanages, and other charities are published in such a form as to be practically without system, and so render it possible for money to be wastefully expended without check, and give rise to other evils of a most serious character. Is it too much to hope that the Committees and Secretaries of these latter general charities will take up this question of uniform accounts, and follow the lead of the hospitals by instituting without delay a most useful and necessary reform?

### The Audit of Charities.

The *Times* recently published some letters advocating the audit of all charitable accounts by chartered accountants. Several letters have appeared, one of which is remarkable from the circumstance that it proves the writer, who signs himself "Honest So Far," to have a full knowledge of the methods pursued by knaves who rob charities, whilst he resents any proposal to institute an effectual audit as an impertinent interference with himself and a reflection upon his honesty. "Honest So Far," as the writer describes himself, declares that, as secretary of one of the oldest charitable institutions in the country, he could defraud his charity without the least difficulty, and without any auditors being able to discover it. He supports this contention by giving instances. These include (1) contributions paid anonymously, with a special request that no names be printed in the report; (2) annual subscriptions paid by cheques drawn to the secretary's order, with a special form of receipt enclosed; and (3) the payment by solicitors by cheque of a legacy to the secretary upon his signing the required form of receipt, no official receipt being required. He scornfully asks, "What chartered accountants need know anything of my having received this money?" He further declares that although the accounts of his charity are audited by one of the best known firms of accountants in the City of London, "I could have misappropriated money over and over again had I wished to do so, and not one of my committee nor the auditors would have been aware of the fact."

The first thing that strikes us in connection with this remarkable letter is the responsibility which will rest upon the Editor of the *Times* if he fails to hand it over to the chairman of the committee of the charity to which the writer belongs. The responsibility of not doing so is increased by the avowal of the writer that he understands how to deal "even with crossed cheques drawn to the order of the charity, and to turn them to his own account without being discovered." It is true that he enclosed his card for the editor's private use only, but an editor has responsibilities, and we venture to think it is clearly the duty of the *Times* to hand over that card and the letter to the chairman of the charity concerned. Each one of the cases mentioned by "Honest So Far," has been brought to our personal knowledge in the case of investigations connected with the defalcations of officials of charities. Despite "Honest So Far's" assertion to the contrary, we know, from long experience, that in every one of these cases it is very easy for the chairman or the auditor to put his hand upon the culprit and to convict him of the theft. We have not space to go into this question in detail here, but we may point out one simple cure for practices of the kind referred to. Each committee should provide that every cheque and remittance intended for the charity is made *payable to the order of the treasurer by name.* They should also have, as at the Westminster Hospital, a small but competent finance committee, which must regularly check every item of receipt and expenditure, comparing such items with those of the previous years; and lastly, they must appoint the ablest financier on the committee as governors' auditor, and associate him with the chartered accountant in the auditing of the accounts each year.

Of course, common honesty used to be, and we believe is still, a common virtue, and we are confident that the whole secretarial world will be in arms against "Honest So Far," whose communication, whether written in joke or in earnest, is quite unworthy of anyone in charge of one of our oldest charitable institutions. We hope his unworthiness will be speedily brought home to the authorities, with the view of placing its affairs in the hands of a responsible secretary.

